# Effect of diabetes medications on the risk of developing dementia, mild cognitive impairment, or cognitive decline: a systematic review and meta‐analysis

**DOI:** 10.1002/alz70860_096280

**Published:** 2025-12-23

**Authors:** Esther K Hui, Naheed Mukadam, Gianna Kohl, Gill Livingston

**Affiliations:** ^1^ University College London, London, United Kingdom; ^2^ Division of Psychiatry, University College London, London, London, United Kingdom; ^3^ University College London, London, London, United Kingdom

## Abstract

**Background:**

Diabetes is a risk factor for dementia, but we do not know whether specific diabetes medications ameliorate this risk. Objective: To systematically review and meta‐analyze such medication's effect on the risk of developing dementia, mild cognitive impairment (MCI), or cognitive decline.

**Method:**

We searched three databases until 21.11.23. We included randomized controlled trials (RCT), cohort, and case‐control studies assessing association between antidiabetic medication and future dementia, MCI, or cognitive decline. We meta‐analyzed studies separately for individual drug classes and their comparators (no medication, placebo, or another drug). We appraised study quality using the Newcastle‐Ottawa Scale and Physiotherapy Evidence Database Scale.

**Result:**

42 studies fulfilled inclusion criteria. Glucagon‐like peptide‐1 receptor agonists (GLP‐1 RA) versus placebo reduced dementia risk by 53% in 3 RCTs (*n* = 15,820, RR=0.47[0.25,0.86]) and 27% in 3 case‐control studies (*n* = 312,856, RR=0.73[0.54,0.99], I^2^=96%). Repaglinide was superior to glibenclamide by 0.8 points on the Mini‐Mental State Examination scale in another RCT. Meta‐analysis of 7 longitudinal studies showed glitazones (*n* = 1,081,519, RR=0.78[0.76,0.81], I^2^=0%) were associated with reduced dementia risk. Metformin (*n* = 999,349, RR=0.94[0.79,1.13], I^2^=98.4%), sulfonylureas (RR=0.98[0.78,1.22], I^2^=83.3%), dipeptidyl peptidase‐IV inhibitors (DPP‐1V) (*n* = 192,802, RR=0.86[0.65,1.15], I^2^=92.9%) and insulin (*n* = 571,274, RR=1.09[0.95,1.25], I^2^=94.8%) were not. Most studies were observational and limited by confounding by indication.

**Conclusion:**

In people with diabetes, RCTs consistently showed GLP‐RAs reduce future dementia risk. Glitazones consistently showed protective effects, without heterogeneity, suggesting potential generalizability of these results. Metformin, sulfonylureas, insulin, and DPP‐1V studies had inconsistent findings. If information is available future studies should consider dosage, severity, and duration.

